# Risk factors for deep vein thrombosis after traumatic lower extremity fracture: A systematic review and meta-analysis

**DOI:** 10.1097/MD.0000000000038439

**Published:** 2024-06-07

**Authors:** Xiaoliang Qian, Yinping Ge, Jian Luo

**Affiliations:** a Department of Orthopaedics, The First People’s Hospital of Linping District of Hangzhou, Hangzhou City, China.

**Keywords:** deep vein thrombosis of lower extremity, fracture, LEDVT, risk factor

## Abstract

**Background:**

The study aimed to predict the risk factors of deep vein thrombosis of lower extremity after traumatic fracture of lower extremity, so as to apply effective strategies to prevent deep vein thrombosis of lower extremity, improve survival rate, and reduce medical cost.

**Methods:**

The English and Chinese literatures published from January 2005 to November 2023 were extracted from PubMed, Embase, Willey Library, Scopus, CNKI, Wanfang, and VIP databases. Statistical analysis was performed using Stata/SE 16.0 software.

**Results:**

A total of 13 articles were included in this paper, including 2699 venous thromboembolism (VTE) patients and 130,507 normal controls. According to the meta-results, 5 independent risk factors can be identified: history of VTE was the most significant risk factor for deep vein thrombosis after traumatic lower extremity fracture (risk ratio [RR] = 6.45, 95% confidence interval [CI]: 1.64–11.26); age (≥60) was the risk factor for deep vein thrombosis after traumatic lower extremity fracture (RR = 1.60, 95% CI: 1.02–2.18); long-term braking was a risk factor for deep vein thrombosis after traumatic lower extremity fracture (RR = 1.52, 95% CI: 1.11–1.93); heart failure was a risk factor for deep vein thrombosis after traumatic lower extremity fracture (RR = 1.92, 95% CI: 1.51–2.33); obesity was a risk factor for deep vein thrombosis after traumatic lower extremity fracture (RR = 1.59, 95% CI: 1.35–1.83).

**Conclusion:**

The study confirmed that the history of deep vein thrombosis, age (60 + years), previous history of VTE, obesity, prolonged bed rest, and heart failure are all associated with an increased risk of VTE. By identifying these significant risk factors, we can more intensively treat patients at relatively high risk of VTE, thereby reducing the incidence of VTE. However, the limitation of the study is that the sample may not be diversified enough, and it fails to cover all potential risk factors, which may affect the universal applicability of the results. Future research should include a wider population and consider more variables in order to obtain a more comprehensive risk assessment.

## 1. Introduction

Venous thromboembolism (VTE) diseases include deep vein thrombosis (DVT) and pulmonary embolism (PE).^[1]^ DVT is an obstructive disease that develops through the formation of abnormal thrombus in the deep venous blood duct, which further obstructs the lower extremity blood vessels and obstructs venous return.^[2]^ It is more common in elderly patients with fractures and bedridden, mainly in the lower extremities.^[2]^ DVT of lower extremity (LEDVT) is a serious multifactorial fatal disease with an annual incidence of 45 to 117 cases/100,000 people,^[3]^ and the mortality rate within 1 month after diagnosis of LEDVT is as high as 6%.^[4]^

However, traumatic lower extremity fractures further increase the incidence of LEDVT.^[5]^ Therefore, we believe that the identification of risk factors for DVT after traumatic lower extremity fractures is very important for clinicians to apply effective strategies to prevent DVT, improve survival rates, and reduce healthcare costs.

To increase our understanding of the risk of DVT after traumatic lower extremity fractures, we collected data from primary studies to identify risk factors for DVT after traumatic lower extremity fractures. Based on these identified risk factors, we can provide more information and improved guidance to clinical traumatic orthopedic workers, thereby reducing morbidity, mortality, and healthcare costs from this disease.

## 2. Materials and methods

### 2.1. Literature search

This meta-analysis was conducted in strict accordance with PRISMA.^[6]^ In this study, English literature and Chinese literature published from January 2005 to November 2023 were extracted from PubMed, Embase, Willey Library, Scopus, CNKI, Wanfang, and VIP databases. The literature search mainly adopts the combination of subject words and unqualified search, and the search formula is: (‘fractures, bone ‘[MeSH Terms]) OR (‘fractures’ [All Fields] AND’ bone’ [All Fields]) OR (‘bone fractures’ [All Fields]) OR (‘bone’ [all fields]) [All Fields] AND (‘fracture’ [All Fields]) OR (‘bone fracture ‘[All Fields] AND’ veins’ [MeSH Terms]) OR (‘veins'All fields] and ‘fracture’ [All Fields]) OR (‘veins’ [all Fields]) OR (‘venous’ [All Fields] AND ‘thrombosis’ [MeSH Terms]) OR (‘thrombosis’ [All Fields]) OR (‘thrombus’ [All Fields] AND ‘risk factors’ [MeSH Terms]) OR (‘risk’ [All Fields] AND ‘factors’ [All Fields]) OR (‘risk factors’ [All Fields]). We then checked the title and abstract before deciding if they could be included in the study. Finally, additional studies were identified from the reference list of key studies.

### 2.2. Inclusion and exclusion criteria

#### 2.2.1. Inclusion criteria

1. Study design type: the study was conducted using case-control or cohort design, and the full text is available.
2. Subjects consisted of adult patients (≥18 years of age) who underwent surgery for traumatic fractures of the lower extremities and reported normal exposure.
3. Results Data on the incidence and risk factors of VTE were reported (such as smoking history, current smoking, age ≥60, long-term braking, VTE history, heart failure, hypertension).
4. Original study with no missing data.

#### 2.2.2. Exclusion criteria

1. Duplicate articles or no full text.
2. Participants were younger than 18 years of age or had received nonsurgical treatment.
3. Research on data missing or errors that cannot be completed and corrected.
4. Lack of outcome indicators required by this study.
5. Letters, case reports, reviews, practice guidelines, etc.
6. All animal experiments.

### 2.3. Data extraction

Two independent investigators collected data on authors, age, each group of patients, sex, number of episodes of DVT (rate), sociodemographic risk factors for VTE after fracture surgery, and study design methods. For studies where no data were directly available in the paper, we contacted the appropriate authors for this information.

### 2.4. Literature quality evaluation

This meta-analysis was conducted by 2 independent investigators who assessed the quality of the studies in our analysis based on the Newcastle–Ottawa Scale (NOS).^[7]^ The NOS scale consisted of three dimensions and eight items: four items for the selection of research objects, one item for inter-group comparability, and three items for outcome measurement. Except for the item of comparability between groups, the maximum score is two points, and the other items can be scored one point, the score range is 0 to 9 points. The higher the total score, the higher the quality of the study. Studies with a score of 6 to 9 are considered to be of high quality and those with a score of 0 to 5 are considered to be of low quality. Studies were scored independently by 2 researchers and any differences between reviewers were resolved by consensus or by a third reviewer.

### 2.5. Statistical analysis

Stata/SE 16.0 software was used in this study. We investigated the incidence and risk factors for VTE after fracture surgery, based on data from studies in the meta-analysis. For the dichotomous variables (i.e., incidence and risk factors for VTE), the number of case events and the number of control patients were extracted from the included studies, and a 95% confidence interval (95% CI) risk ratio (RR) was calculated. *Q* test was used to test the heterogeneity between studies. If *I*^2^ < 50% and *P* > .1, the heterogeneity between studies was small, and fixed effects model was used. Otherwise, the random effects model is used to calculate the combined effect size. Forest maps were used to describe the statistical results of the meta-analysis.

## 3. Results

### 3.1. Literature screening results

**Figure 1.**
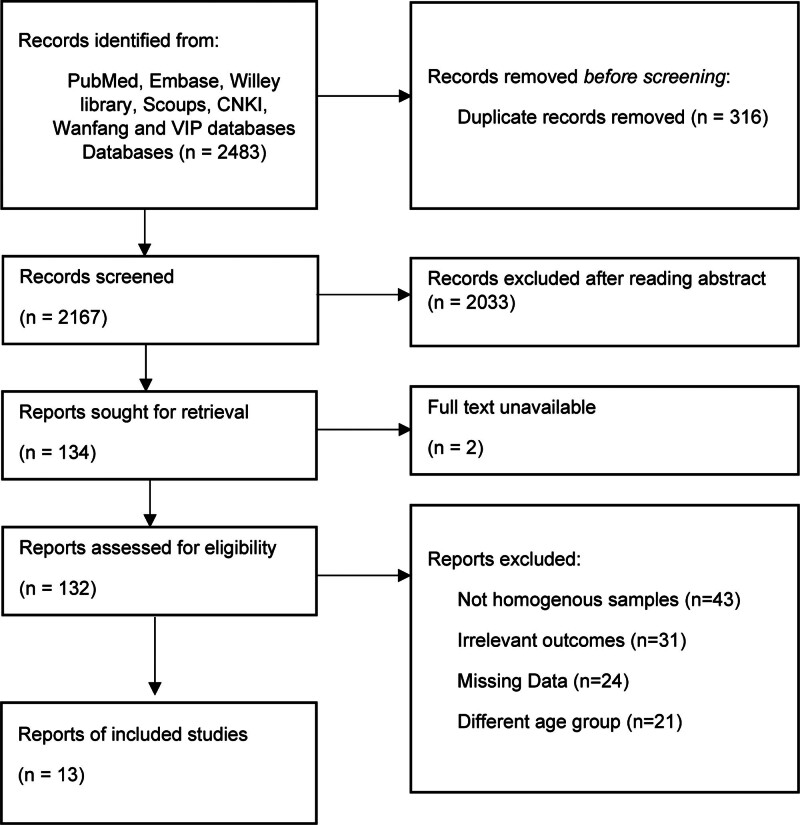
Literature screening flowchart.

A total of 2483 studies were extracted from the seven databases through the above retrieval methods, and 2167 original studies were extracted after the exclusion of duplicate articles. By reviewing the title, keywords, and abstract, 134 of the studies were identified as likely to be relevant to the research topic. We searched further for the full text of these studies and got the full text of 92 studies in total. We excluded another 121 studies based on inclusion and exclusion criteria. Finally, a total of 13 studies^[8–20]^ were included in this meta-analysis. The literature screening process is shown in Figure 1.

### 3.2. Basic features included in the study

The 13 included literatures^[8–20]^ are all original studies. This included 2699 VTE patients and 130,507 normal healthy controls. The basic features of the included studies are shown in Table 1.

**Table 1:** Basic features included in the study.

First author	Year	Country	Study design	Sample size (case)	Sample size (control)
Wahlsten et al^[8]^	2015	Denmark	Cohort	594	57,025
Mantilla et al^[9]^	2003	USA	Case–control	116	116
Yang^[10]^	2010	China	Case–control	58	457
Akpinar et al^[11]^	2013	Turkey	Case–control	55	1251
Pedersen et al^[12]^	2010	Denmark	Case–control	1390	66,079
Park et al^[13]^	2015	Korea	Cohort	38	863
Riou et al^[14]^	2007	France	Case–control	178	2582
Gu^[15]^	2007	China	Case–control	18	84
Wang^[16]^	2013	China	Case–control	52	51
Long^[17]^	2013	China	Case–control	73	72
Tan Z et al^[18]^	2021	China	Case–control	29	687
Ding K et al^[19]^	2022	China	Case–control	51	806
Peng J et al^[20]^	2023	China	Case–control	47	434

### 3.3. Quality evaluation of the included literature

The quality of the literatures was evaluated based on the NOS, and the quality evaluation results of the included literatures are shown in Table 2.

**Table 2:** Quality evaluation of included studies.

First author [reference number]	Quality evaluation score (NOS)	Selection of research subjects				Comparability between groups	Measurement of outcome		
		Representative of experimental group (AD/MCI)	Representative of control group	Definition of experimental group (AD/MCI)	Definition of control group		Outcome index measurement	Follow-up time	Follow-up of integrity
Wahlsten et al^[8]^	9	Good	Good	Clarity	Good	Good	Low risk	Long	Good
Mantilla et al^[9]^	8	Good	Good	Uncertainty	Clarity	Good	Low risk	Long	Good
Yang^[10]^	6	Uncertainty	Good	Clarity	Uncertainty	Uncertainty	Low risk	Long	Good
Akpinar et al^[11]^	8	Good	Good	Clarity	Clarity	Uncertainty	Low risk	Long	Good
Pedersen et al^[12]^	7	Uncertainty	Good	Clarity	Clarity	Uncertainty	Low risk	Long	Good
Park et al^[13]^	7	Good	Uncertainty	Clarity	Clarity	Good	Low risk	Long	Uncertainty
Riou et al^[14]^	5	Good	—	Clarity	—	Uncertainty	Low risk	Long	Uncertainty
Gu^[15]^	6	Good	—	Clarity	—	Uncertainty	Low risk	Long	Good
Wang^[16]^	9	Good	Good	Clarity	Clarity	Uncertainty	Low risk	Long	Good
Long^[17]^	6	Good	—	Clarity	—	Uncertainty	Low risk	Long	Good
Tan Z et al^[18]^	7	Good	—	Clarity	Clarity	Uncertainty	Low risk	Long	Good
Ding K et al^[19]^	6	Uncertainty	Good	Clarity	Clarity	Uncertainty	Low risk	Long	Good
Peng J et al^[20]^	7	Uncertainty	Good	Clarity	Clarity	Uncertainty	Low risk	Long	Good

NOS = Newcastle–Ottawa Scale.

### 3.4. Meta-analysis results and sensitivity analysis

#### 3.4.1. The relationship between current smoking, smoking history, and LEDVT after traumatic fracture of lower extremity

**Figure 2.**
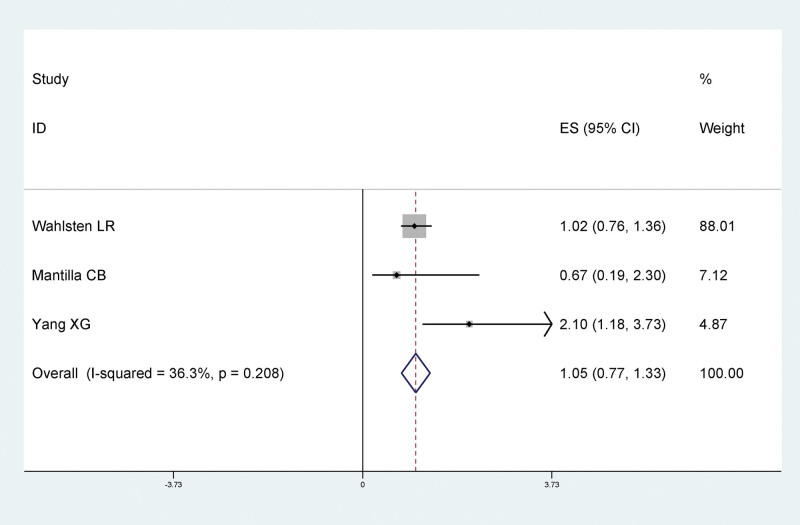
Forest plot of the relationship between current smoking and DVT after traumatic lower extremity fracture. DVT = deep vein thrombosis.

**Figure 3.**
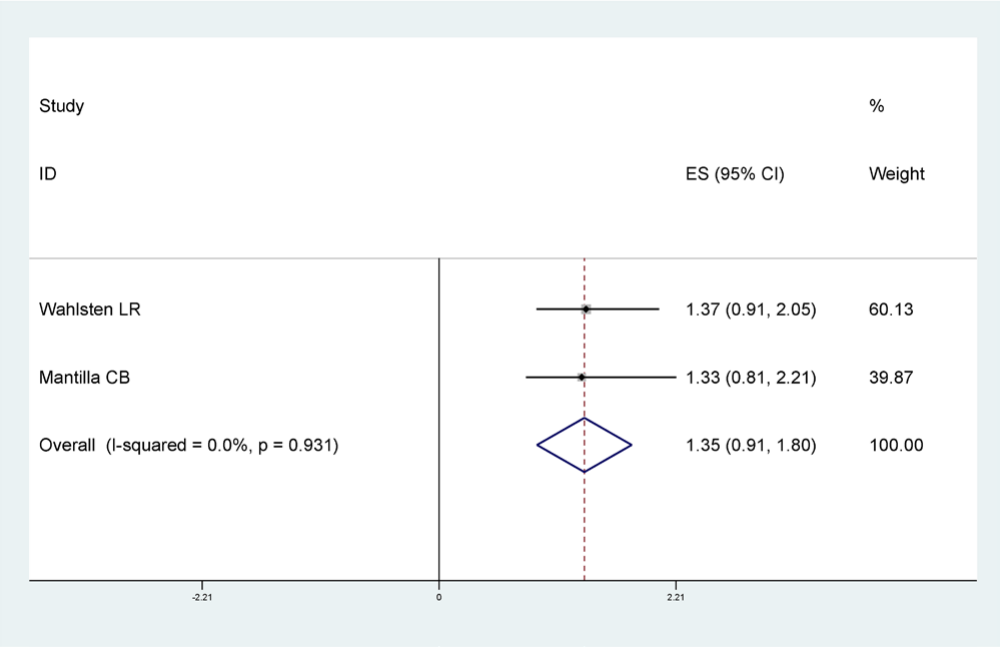
Forest plot of relationship between smoking history and LEDVT after traumatic fracture of lower extremity. LEDVT = deep vein thrombosis of lower extremity.

In order to differentiate the effects of previous and current smoking on LEDVT after traumatic fracture of lower extremity, we analyzed current smoking and and smoking history separately. Results of meta-analysis showed that there was no significant statistical relationship between smoking and LEDVT after traumatic fracture of lower extremity, and smoking was not a risk factor for LEDVT after traumatic fracture of lower extremity (RR = 1.05, 95% CI: 0.77–1.33), as shown in Figure 2. There was no statistically significant relationship between smoking history and LEDVT after traumatic fracture of lower extremity, and smoking history was not a risk factor for LEDVT after traumatic fracture of lower extremity (RR = 1.35, 95% CI: 0.91–1.80), as shown in Figure 3, suggesting that past smoking and current smoking had little effect on LEDVT after traumatic fracture of lower extremity.

#### 3.4.2. The relationship between age (≥60) and LEDVT after traumatic fracture of lower extremity

**Figure 4.**
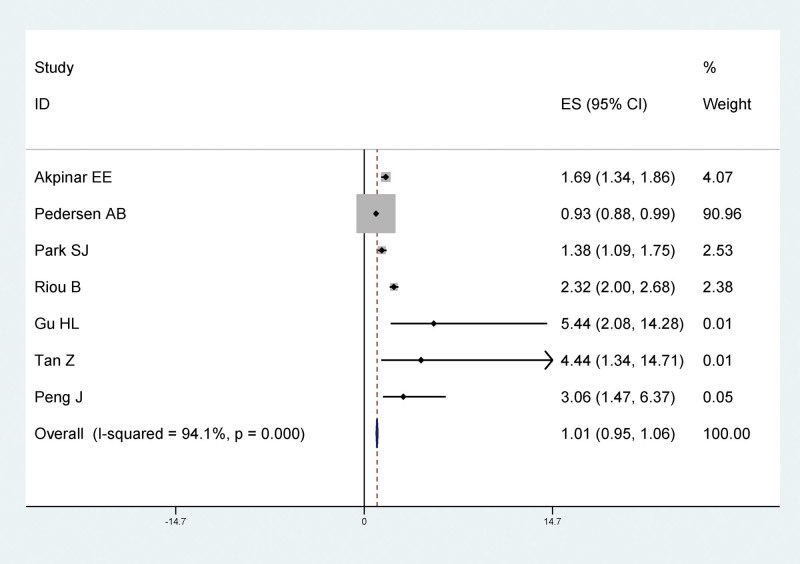
Forest plot of the relationship between age (≥60) and LEDVT after traumatic fracture of lower extremity. LEDVT = deep vein thrombosis of lower extremity.

The results of meta-analysis showed that age (≥60) had a statistically significant relationship with LEDVT after traumatic lower extremity fracture, and age (≥60) was a risk factor for LEDVT after traumatic lower extremity fracture (RR = 1.01, 95% CI: 0.95–1.06), as shown in Figure 4.

#### 3.4.3. Long-term immobilization and LEDVT after traumatic fracture of the lower extremity

**Figure 5.**
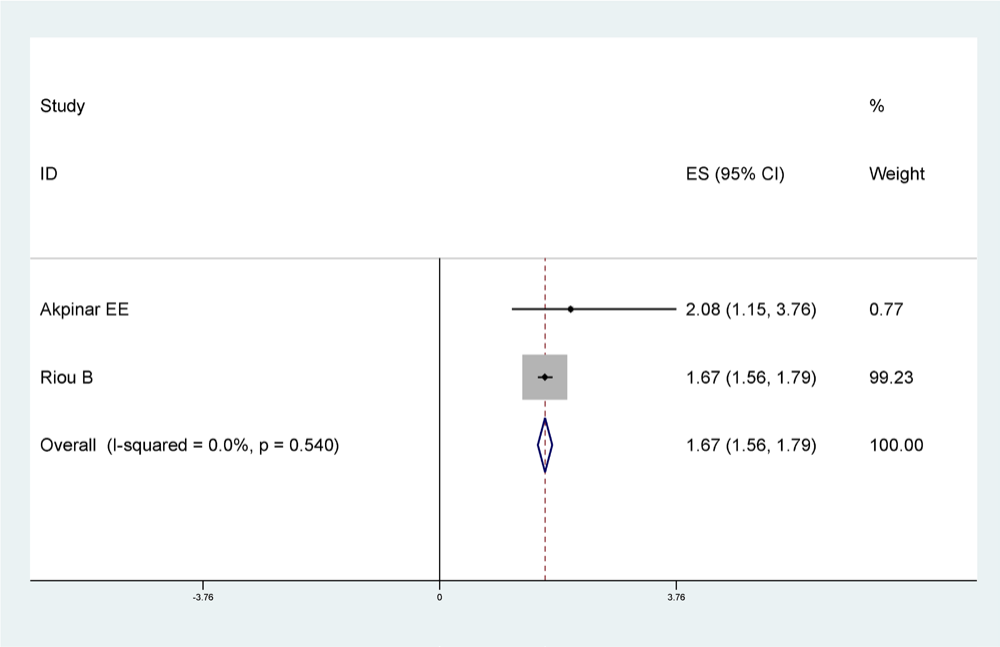
Forest plot of relationship between long-term braking and LEDVT after traumatic fracture of lower extremity. LEDVT = deep vein thrombosis of lower extremity.

Results of meta-analysis showed that long-term braking had a statistically significant relationship with LEDVT after traumatic fracture of lower extremity. Long-term braking was a risk factor for LEDVT after traumatic fracture of lower extremity (RR = 1.67, 95% CI: 1.56–1.79), as shown in Figure 5.

#### 3.4.4. The relationship between history of VTE and LEDVT after traumatic fracture of lower extremity

**Figure 6.**
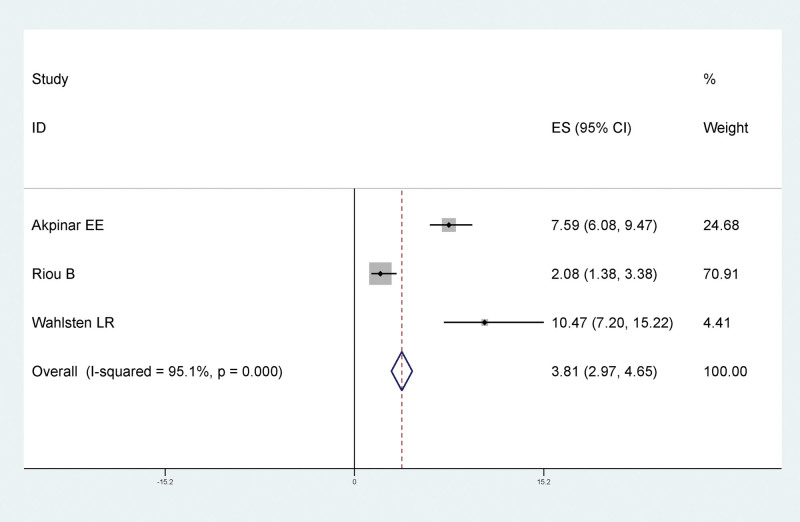
Forest plot of the relationship between VTE history and LEDVT after traumatic fracture of lower extremity. LEDVT = deep vein thrombosis of lower extremity, VTE = venous thromboembolism.

The results of meta-analysis showed that there was a statistically significant relationship between VTE history and LEDVT after traumatic lower extremity fracture, and VTE history was a risk factor for LEDVT after traumatic lower extremity fracture (RR = 3.81, 95% CI: 2.97–4.65), as shown in Figure 6.

#### 3.4.5. The relationship between heart failure and LEDVT after traumatic fracture of lower extremity

**Figure 7.**
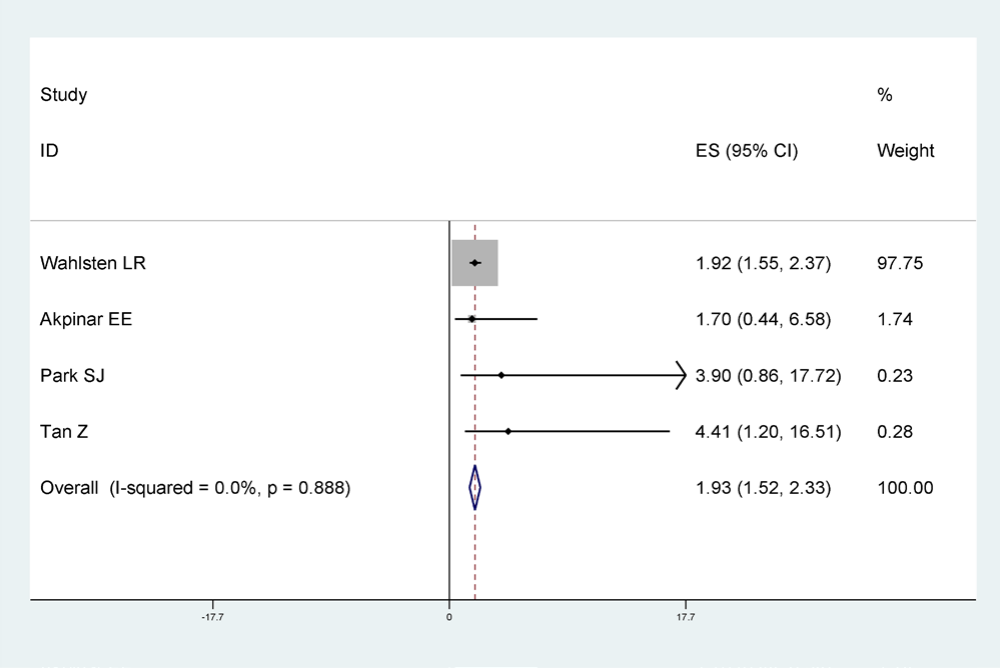
Forest plot of relationship between heart failure and LEDVT after traumatic fracture of lower extremity. LEDVT = deep vein thrombosis of lower extremity.

Results of meta-analysis showed that there was a statistically significant relationship between heart failure and LEDVT after traumatic fracture of lower extremity, and heart failure was a risk factor for LEDVT after traumatic fracture of lower extremity (RR = 1.93, 95% CI: 1.52–2.33), as shown in Figure 7.

#### 3.4.6. The relationship between hypertension and LEDVT after traumatic fracture of lower extremity

**Figure 8.**
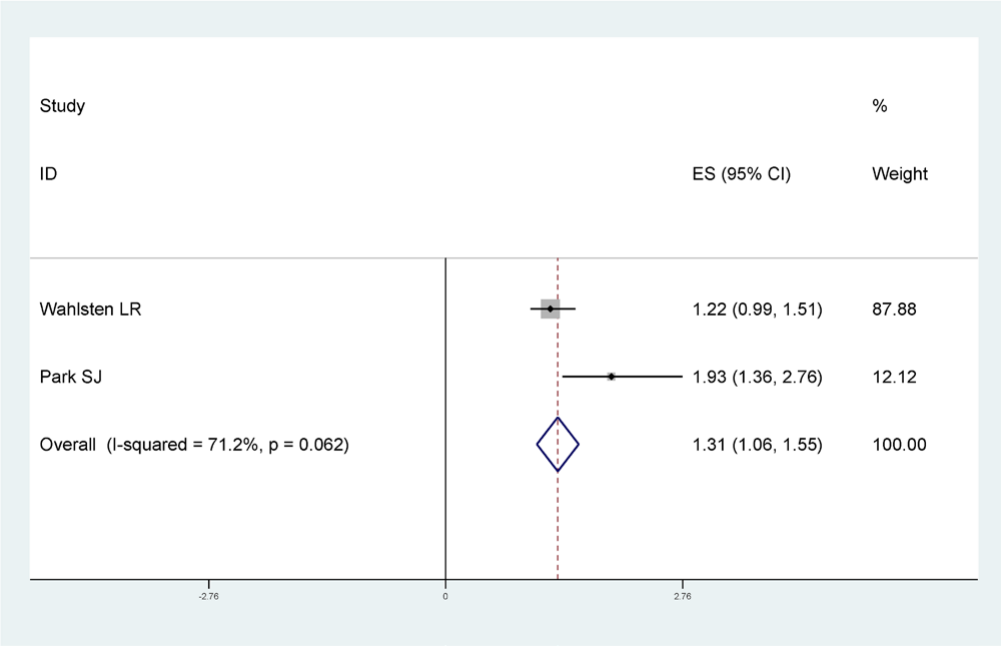
Forest plot of the relationship between hypertension and LEDVT after traumatic fracture of lower extremity. LEDVT = deep vein thrombosis of lower extremity.

Results of meta-analysis showed that there was no significant statistical relationship between hypertension and LEDVT after traumatic fracture of lower extremity (RR = 1.31, 95% CI:1.06–1.55), as shown in Figure 8.

#### 3.4.7. Obesity and LEDVT after traumatic fracture of lower extremity

**Figure 9.**
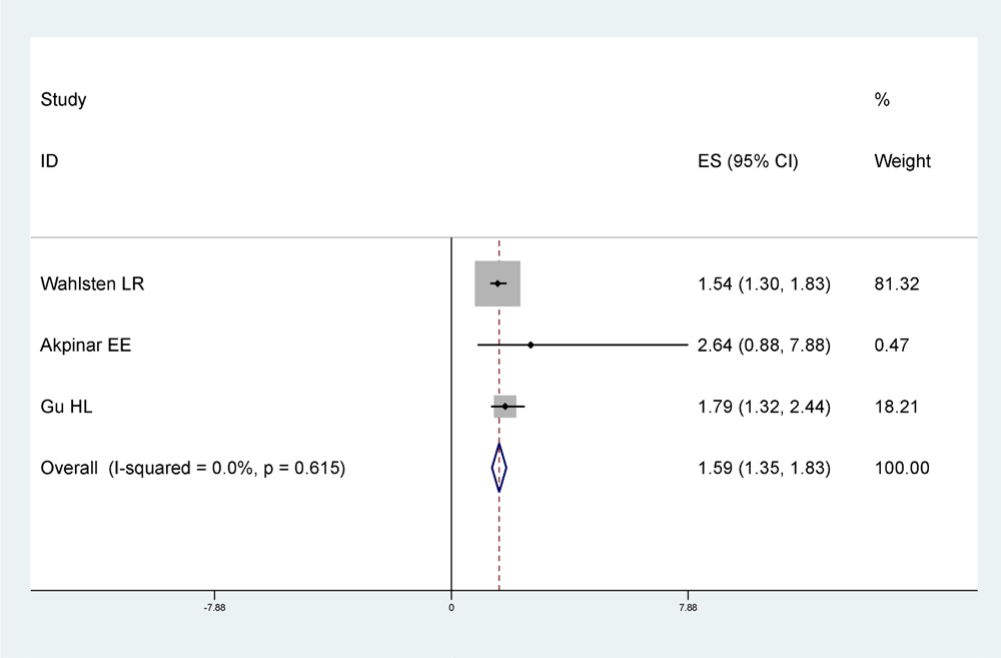
Forest plot of the relationship between obesity and LEDVT after traumatic fracture of lower extremity. LEDVT = deep vein thrombosis of lower extremity.

Results of meta-analysis showed that there was a statistically significant relationship between hypertension and LEDVT after traumatic fracture of lower extremity, and obesity was a risk factor for LEDVT after traumatic fracture of lower extremity (RR = 1.59, 95% CI: 1.35–1.83), as shown in Figure 9.

#### 3.4.8. The relationship between single-site traumatic lower extremity fracture and LEDVT after fracture

**Figure 10.**
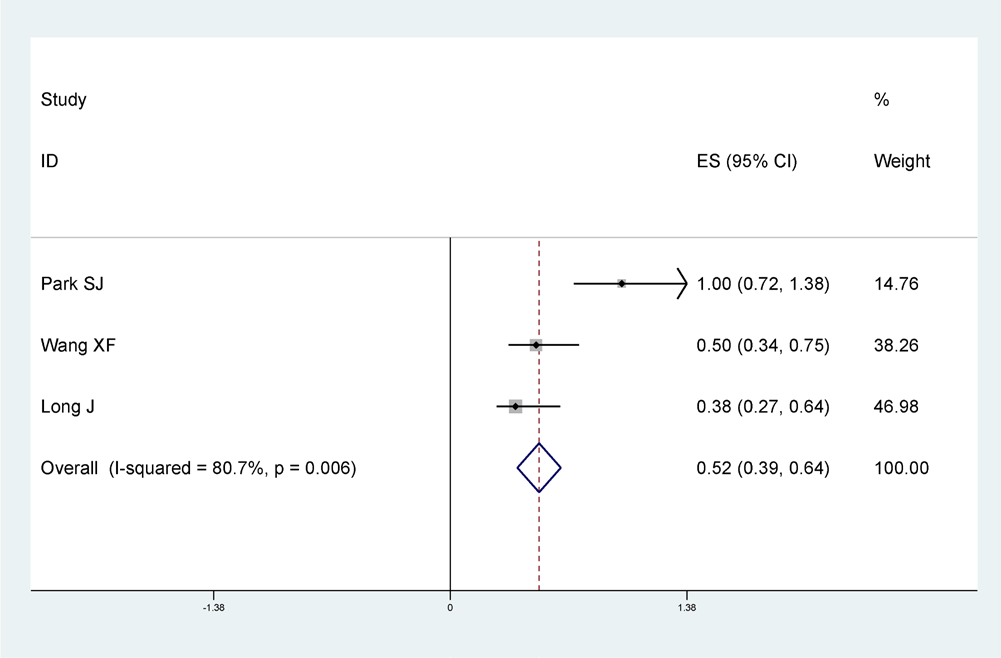
Forest plot of relationship between single-site traumatic lower extremity fracture and postfracture LEDVT. LEDVT = deep vein thrombosis of lower extremity.

Results of meta-analysis showed that there was a statistically significant relationship between single-site traumatic lower extremity fractures and LEDVT after fracture, and single-site fractures were protective factors for LEDVT after traumatic fracture (RR = 0.52, 95% CI: 0.39–0.64), as shown in Figure 10.

#### 3.4.9. Multisite traumatic lower extremity fracture and LEDVT after fracture

**Figure 11.**
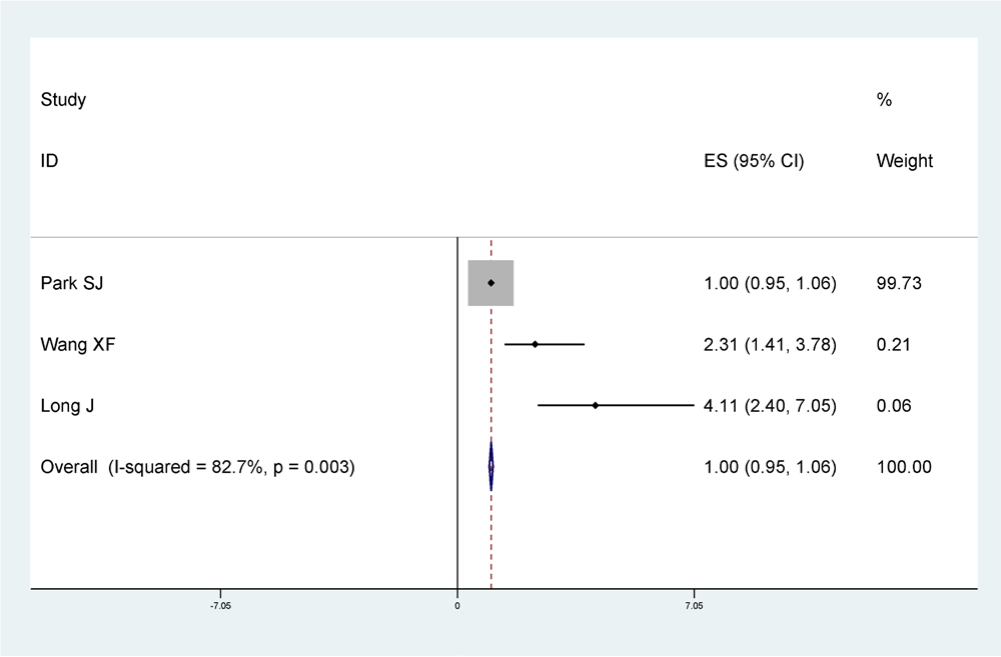
Forest plot of the relationship between multiple site traumatic lower extremity fracture and LEDVT after fracture. LEDVT = deep vein thrombosis of lower extremity.

Meta-analysis results showed that there was no significant statistical relationship between multisite traumatic lower extremity fractures and LEDVT after fracture, and multisite fractures were not risk factors for LEDVT after traumatic fracture (RR = 1.00, 95% CI: 0.95–1.06), as shown in Figure 11.

## 4. Discussion

VTE, including asymptomatic and symptomatic DVT and pulmonary thromboembolism (PE), is a potentially serious complication in patients with traumatic lower extremity fracture.^[21]^ It has been reported that the incidence of DVT and PE in these patients is 8%–70% and 1%–10%.^[1]^ In addition, compared with patients with traumatic fractures without VTE, the medical costs of patients with VTE are 10 times higher and the length of hospital stay is more than twice as long.^[22]^ Therefore, we should implement effective strategies in medical and surgical patients to identify risk factors for VTE and then prevent the occurrence of VTe-related morbidity and mortality.

Identifying risk factors for VTE is a critical and challenging task because there are a large number of potential VTE risk factors of concern. Several studies focusing on patient demographic factors, such as increased body mass index and past VTE history, have found consistent associations between these factors and increased VTE.^[21,23]^ However, for other risk factors, including smoking status, age, and hypertension, there are still controversial results.^[1,3,21,24]^ These may be attributed to several design issues: including small sample sizes, unrepresentative selected patient populations, recruitment of patients from a single institution and inadequate control for confounding factors. We believe that it is very important for clinical workers to identify risk factors for LEDVT after traumatic fracture of lower extremity to apply effective strategies to prevent the occurrence of LEDVT, improve survival rate and reduce medical costs.^[22]^

In this meta-analysis of risk factors of DVT after traumatic lower extremity fracture, we identified 5 significant independent risk factors: previous VTE, advanced age (≥60 years old), long-term immobility, heart failure, and obesity. These results have an important impact on clinical strategy and patient management. First of all, the history of VTE as the strongest risk predictor reminds us to take more active monitoring and preventive measures in patients with this history. In addition, although old age is an unchangeable risk factor, it emphasizes the necessity of implementing more comprehensive risk assessment and prevention strategies among elderly patients. Second, as modifiable risk factors, long-term immobility, heart failure, and obesity pointed out the key intervention points to prevent DVT. For patients with long-term bed rest or limited mobility, active rehabilitation, and appropriate drug intervention are important measures to reduce DVT. In addition, the comprehensive management strategy for heart failure and obesity cannot be ignored to reduce the risk of DVT. Although current smoking, smoking history, and hypertension are usually considered as risk factors for many diseases, they are not significantly related to the risk of DVT in this study. This may reflect the heterogeneity between different populations and research methods. Therefore, future research should further explore the potential relationship between these factors and DVT. In addition, single-site fracture seems to reduce the risk of DVT compared with multisite fracture, which may be related to the degree of physiological stress and inflammatory reaction. This finding suggests that we should pay more attention to the influence of fracture type and degree on the risk of DVT in the future.

To sum up, the results of this meta-analysis not only provide practical clinical guidance for the prevention and treatment of DVT after traumatic lower extremity fracture, but also reveal many directions for future research, so as to understand and manage the risks of DVT more accurately.

## Author contributions

**Conceptualization:** Xiaoliang Qian.

**Data curation:** Xiaoliang Qian, Yinping Ge, Jian Luo.

**Writing—original draft:** Xiaoliang Qian, Yinping Ge, Jian Luo.

**Writing—review & editing:** Xiaoliang Qian, Yinping Ge, Jian Luo.

**Project administration:** Yinping Ge.

**Formal analysis:** Jian Luo.
